# Statistical validation of wavelet transform coherence method to assess the transfer of calf muscle activation to blood pressure during quiet standing

**DOI:** 10.1186/1475-925X-12-132

**Published:** 2013-12-23

**Authors:** Amanmeet Garg, Da Xu, Andrew P Blaber

**Affiliations:** 1 School of Engineering Science, Simon Fraser University, 8888 University Drive, Burnaby, BC, Canada; 2 Aerospace Physiology Laboratory, Department of Biomedical Physiology and Kinesiology, Simon Fraser University, 8888 University Drive, Burnaby, BC, Canada

**Keywords:** Wavelet transform coherence, Calf muscle EMG, Quiet standing, Blood pressure, Posture control, Skeletal muscle pump

## Abstract

**Background:**

Continuous and discrete wavelet transforms have been established as valid tools to analyze non-stationary and transient signals over Fourier domain methods. Additionally, Fourier transform based coherence methods provide aggregate results but do not provide insights into the changes in coherent behavior over time, hence limiting their utility.

**Methods:**

Statistical validation of the wavelet transform coherence (WTC) was conducted with simulated data sets. Time frequency maps of signal coherence between calf muscle electromyography (EMG) and blood pressure (BP) were obtained by WTC to provide further insight into their interdependent time-varying behavior via the skeletal muscle pump during quiet stance. Data were collected from healthy young males (n = 5, 19–28 years) during a quiet stance on a balance platform. Waveforms for EMG and BP were acquired and processed for further analysis.

**Results:**

Low values of bias and standard deviation (< 0.1) were observed and the use of both simulated and real data demonstrated that the WTC method was able to identify time points of significant coherence (> Threshold) and objectively detect existence of interdependent activity between the calf muscle EMG and blood pressure.

**Conclusions:**

The WTC method effectively identified the presence of linear coupling between the EMG and BP signals during quiet standing. Future studies with more human data are needed to establish the exact characteristics of the identified relationship.

## Background

The skeletal muscle pump as we understand it, pumps venous blood pooled in lower limbs back to the heart through contractions under varied circumstances [1]. This activation has been shown to be essential to maintain venous return and blood pressure (BP) during standing [2] and after exercise [3].

This mechanism is indicative of a possible baroreflex-induced interaction between BP regulation and lower limb muscle activation. The potential interaction between BP and postural sway (as an indicator of skeletal muscle pump activity) has been shown in terms of their relationships to lower limb and trunk discomfort [4]. In a previous study we investigated the direct interaction between BP and postural sway [5] through classical coherence analysis, a method which has been extensively applied to physiological time series that are generated by a combination of complex interactions. We observed that mediolateral (ML) postural sway and BP were related with significant coherence in the frequency range of 0.01 – 0.1 Hz. This frequency band was within the range previously reported between postural sway and calf muscle electromyogram (EMG) (0 – 0.2 Hz) [6]. This provided motivation for more direct evidence of blood pressure mediated skeletal muscle pump activation through the relation between EMG and BP signals.

The BP signal has been extensively studied for the analysis of baroreflex sensitivity, respiratory sinus arrhythmia, and other cardiovascular conditions [7]. On the other hand, studies of EMG signals from the lower leg muscles have been largely focused on exercise and posture assessments [6] with minimal focus on their relation with BP changes. Both EMG and BP signals have been identified as non-linear and non-stationary in nature [8,9], as they are both time and pulse dependent, thus requiring a more sophisticated approach than the classical Fourier based coherence analyses. The activation of the skeletal muscle pump (pulse) to improve venous return during conditions of declining blood pressure is an interaction of complex nature presenting non-trivial structure due to the number of regulatory mechanisms in the blood flow control [10].

In this study we chose to use the wavelet transform, a method which has been used in many forms to analyze physiological signals. Discrete wavelet transforms (DWT) and continuous wavelet transforms (CWT) have been used for feature extraction analysis of EMG signal [11-13] and applied to investigations of low frequency BP fluctuations in rats [14], peripheral blood circulation oscillations [15], and vasovagal syncope [16]. The use of both DWT and CWT in cardiovascular signal analysis has been reviewed in detail by Addison [17].

The coherence function is a method used to assess the existence and strength of linear coupling between two signals in the frequency domain [18]. Classical coherence and correlation methods have been used to investigate the relationship between signals; however, signal stationarity is assumed. This stationarity assumption can potentially overshadow the dynamic characteristics required for continuous physiological adjustments to maintain homeostasis. Therefore, Fourier based coherence and spectral analysis methods including short-time Fourier transform are of limited utility. The wavelet transform coherence (WTC) method is a known signal analysis tool for random-like deterministic signals created by complex and not fully understood mechanisms. It has been investigated and applied in cardiovascular analysis [19] with the aim of understanding the response of the autonomic control system to induced orthostatic stress, an area related to the proposed research.

Wavelet transform coherence is used to find transient correlations between signals. The most common use of wavelet coherence is to find correlated areas between signals that are uncorrelated for most of the time. The WTC method provides information on the strength of the relationship as a time frequency map. In this way, related signal features can be obtained over specific frequency zones and time points. Desired resolution can be obtained simultaneously for each signal feature; higher temporal resolution for higher frequencies, and higher spatial resolution for lower frequencies.

The scope of this work was to investigate the applicability of the WTC method as a tool for identification of presence of relationship between the cardiovascular and postural control systems. The preliminary attempt was made utilizing the classical coherence method to find the existence of the relationship as per the previous work [5]. Bias, standard deviation, and coherence threshold were calculated in the frequency range of interest using simulated signals in order to evaluate the baseline characteristics of the method independent of human variability for the signals under investigation. Finally, the WTC estimator was applied to real physiological signals acquired from the two systems in a quiet stand test.

## Methods

### Wavelet transform

We applied the Morlet wavelet to the WTC estimation in our study since it is a commonly used mother wavelet for the analysis of physiological signals [17,19]. A description of its application in this study is presented below while more detailed discussion of the WTC method can be found in previously published work by Torrence and Compo [20] and Grinsted et al. [21].

Briefly, the discrete form of the CWT is shown in Equations 1 and 2 where *x*_*n*_ is the digitized time series with time step *δt*, n = 1,.,., N, s represents scale and Ψ is the scaled and translated mother wavelet.

(1) $$W_{n}\left(s\right)=\;\sum_{n'=0}^{N-1}x_{n'}\Psi^{*}\left[\frac{\left(n'‒n\right)\mathrm{\delta t}}{s}\right]$$

(2) $$\Psi \left[\frac{\left(n'‒n\right)\mathrm{\delta t}}{s}\right]={\left(\frac{\mathrm{\delta t}}{s}\right)}^{1/2}\Psi_{0}\left[\frac{\left(n'‒n\right)\mathrm{\delta t}}{s}\right]$$

The Morlet coefficient, *ω*_0_, defines the balance between frequency and time resolution where *ω*_0_>6 is the minimum requirement as per the admissibility condition [22]. For our analyses and frequency resolution requirements, we tested the coherence estimator in the range 6 < *ω*_0_ < 30 for its statistical acceptance.

For the mother wavelet, the scale to frequency transformation (Equation 3) was defined through the Fourier wavelength [20]. The scale to frequency conversion enabled the creation of a spectrogram as a time frequency map of coherence amplitude against a less intuitive time scale map.

(3) $$\lambda =\frac{1}{f}=\frac{4\mathrm{\pi s}}{\omega_{0}+\sqrt{2+\omega_{0}^{2}}}$$

Based on previous work in our laboratory where we first investigated the frequency dependent relationship between postural sway and BP [5], analysis with the WTC estimator was conducted for three frequency bands which encompass the frequency ranges commonly associated with cardiovascular regulation [19], namely, High Frequency (HF) band 0.5 – 0.1 Hz; Low Frequency (LF) band 0.1 – 0.05 Hz; and Very Low Frequency (VLF) band 0.05 – 0.01 Hz. The coherence output in the time frequency map was averaged over each respective frequency band to obtain the three band coherence estimates.

The squared wavelet coherence estimator was defined as the squared absolute value of the smoothed cross- wavelet spectrum $\left(W_{n}^{\mathrm{xy}}\right)$, normalized by the smoothed power spectrum of the two signals $\left(W_{n}^{x},W_{n}^{y}\right)$ (Equation 4).

(4) $${\hat{C}}_{n}^{2}=\frac{{\left|\langle W_{n}^{\mathrm{xy}}\left(s\right)\;.\;s^{-1}\rangle \right|}^{2}}{\langle \left|W_{n}^{\mathrm{xx}}\left(s\right)\right|\;.\;s^{-1}\rangle \langle \left|W_{n}^{\mathrm{yy}}\left(s\right)\right|\;.\;s^{-1}\rangle }$$

The wavelet power density estimator of *x*_*n*_ is defined as $W_{n}^{\mathrm{xx}}\left(s\right)=W_{n}^{x}W_{n}^{x*}$ where, $W_{n}^{x*}$ was the complex conjugate of the wavelet coefficient $W_{n}^{x}$ (Equation 1). The cross wavelet transform of two time series, *x*_*n*_ and *y*_*n*_, was defined as $W_{n}^{\mathrm{xy}}=W_{n}^{x}\left(s\right)W_{n}^{y*}\left(s\right)$. The symbol 〈·〉 in Equation 4 was the smoothing operator as defined by Torrence and Webster [23].

### Statistical validation for the WTC method

Statistical validation of the new method for application to physiological signals was needed to establish the participant independent baseline characteristics. Simulated signals were generated to closely resemble the real signal in the analysis. As our analysis involves two different types of signals, we independently simulated the EMG and systolic blood pressure (SBP) signals; Rowell [1] demonstrated that both the transition from supine to upright stance and induced postural sway have a greater effect on systolic blood pressure than diastolic or mean arterial pressure. Similar to Bonato and colleagues [24], the myoelectric signal obtained from surface electrodes was modeled as a filtered noise signal. Both Gaussian and Laplacian noise signals were considered in the simulation to take into account the effects of muscle contraction levels on EMG signal distribution. That is, EMG recorded at low contraction levels has super-Gaussian distribution (e.g., Laplacian distribution) and tends to be Gaussian with increasing contraction level [25]. A shaping filter was used, as suggested by Stulen and Deluca [26], with the following transfer function (Equation 5):

(5) $$H\left(f\right)=\frac{k^{2}f_{h}^{4}f^{2}}{\left(f^{2}+f_{l}^{2}\right){\left(f^{2}+f_{h}^{2}\right)}^{2}}$$

Where: *f*_*l*_*=* bandpass low cut off frequency, *f*_*h*_*=* bandpass high cut off frequency, and *k* = 1.699/*f*_*h*_*k.*

The EMG signal was synthesized using the transfer function in Equation 5 with the low and high cutoff frequency at 0.01 and 0.5 Hz, respectively. As the SBP signal variation does not have a consistent pattern, the SBP signal was modeled as a bandpass filtered white noise signal in the range 0.01 – 0.5 Hz to simulate an arbitrary SBP signal. For all simulated signals (EMG, SBP) 2400 data points were generated at a sampling frequency of 10Hz to create a data length equal to 4 minutes.

The theoretical coherence estimation was based on the model of a single input single output (SISO) linear time invariant (LTI) system [27,28]. The theoretical coherence between X(t) and Y(t) was given by:

(6) $$\gamma^{2}\left(f\right)=\frac{1}{1+G_{\mathrm{NN}}\left(f\right)/G_{\mathrm{XX}}\left(f\right)}$$

Where *G*_*XX*_ (*f*) and *G*_*NN*_ (*f*) were the spectral density functions of the input signal, X(t), and the noise N(t) that was added to get the output signal, Y(t), respectively [29]. In order to apply the model to our time-frequency analysis, we used the wavelet power spectral density function in Equation 6:

(7) $$\gamma_{n}^{2}\left(s\right)=\frac{1}{1+W_{n}^{\mathrm{NN}}\left(s\right)/W_{n}^{\mathrm{XX}}\left(s\right)}$$

Equation 7 shows that the theoretical coherence is effectively determined by the signal to noise ratio (SNR) of the system given by the denominator and vice versa [27,28], which is further controlled through the variance of the added noise relative to the input.

The simulated data were then used to estimate the coherence through WTC which was compared to the theoretical coherence to determine signal bias and standard deviation. Bias of a measurement reflects the tendency towards a particular value in the range of measurement and standard deviation measures the spread of the estimation from the mean value. Under an ideal scenario one would expect to have low values of bias and standard deviation, and choose appropriate parameters for the estimators. In accordance with the SISO system model for most physiological systems [27], the output signal, Y(t), was obtained by the addition of zero mean Gaussian white noise to the input X(t) (simulated signals) with a variance equivalent to that calculated with the SNR through Equation 6.

The bias and the standard deviation (SD) estimates for the modulus of the transfer function for the SISO system have previously been defined by Pinna and Maestri [27], these estimates were adapted for the WTC method and new bias (Equation 8) and standard deviation (Equation 9) estimates were obtained:

(8) $$\mathrm{bias}\left(k\right)≅\left(\frac{1}{N}\sum^{N}C^{2}\left(k\right)\right)-k=\bar{C^{2}\left(k\right)}-k$$

(9) $$\mathrm{SD}\left(k\right)≅\sqrt{\frac{1}{N-1}\sum^{N}{\left(C^{2}\left(k\right)-\bar{C^{2}\left(k\right)}\right)}^{2}}$$

where k was the theoretical coherence level (γ^2^) from 0.05 to 0.95 in 0.1 steps, and C^2^(k) was the calculated WTC value for the signal pair associated with each theoretical coherence level k (through SNR in Equation 6). This procedure was repeated for the different wavelet coefficients *ω*_0_ = 6, 10, 15, 20, 30. The bias and SD measures were calculated as an average over the values obtained for 1000 mutually independent synthesized input/output signal pairs.

As a final step, the threshold of the coherence estimator was determined. The threshold defines the value above which the coherence will be considered significant, and the two signals to have linearly dependent behavior at that time point. The coherence output for two completely uncoupled signals provides information about the significance threshold values for the particular coherence estimator.

To find the threshold for the WTC estimator, signals for SBP and EMG were synthesized as defined above. Using the SISO system model, the output signals were obtained with added white noise, but the variance was kept at a level that gave a SNR < <1, which provided a theoretical band coherence value close to zero (Equation 7). The input/output pairs were then created and checked for the threshold of WTC for different values of *ω*_0._

The band coherence was estimated between each input/output pair and averaged over 1000 iterations to give a coherence time series; and the empirical sampling distribution (frequency histogram) was computed for each frequency band. The threshold for zero coherence, T(f), was set at the 100(1-α) percentile of the coherence sampling distribution, where α is the significance level of the statistical test kept at 95% confidence or 0.05 [30].

### Data collection

Based on the coherence threshold provided by the simulation, data from five participants were collected to investigate *in vivo* interactions between EMG and blood pressure. The protocol followed in the present study was approved by the office of research ethics of the Simon Fraser University to be of minimal risk to the participants. All participants provided written informed consent prior to starting the experiment.

Experimental data were collected following the protocol described by Blaber et al. [5]. Additionally, bilateral lower leg EMG was performed for four leg muscles: tibialis anterior, medial gastrocnemius, lateral gastrocnemius, and medial soleus. Transdermal differential recording of signals was performed using an 8-channel EMG system, (Myosystem 1200, Noraxon Inc., Arizona, USA). For signal transduction, Ag/AgCl dual electrodes (2 cm inter-electrode distance) were used at the muscle sites, and a single Ag/AgCl electrode was placed at the right lateral malleolus as a reference electrode. Electrocardiography (ECG) signals were acquired (LifePak 8, Medtronic Inc, Minnesota, USA) using the Lead II configuration of ECG electrode placement. Blood pressure signals were acquired by photoplethysmography using a finger cuff electrode (Finapres, *Ohmeda 2300* Ohmeda, Ohio, USA). The postural sway data, in terms of the coordinates of the COP of the body, were calculated from the force and moment data collected with a force platform (Accusway, Advanced medical technologies Inc, MA, USA).

All data were acquired using a 32-analog input channel data acquisition card and Labview 8.2 software (National Instruments Inc., TX, USA) with a sampling rate of 1000 Hz. Data were filtered with a Butterworth filter of fourth order with a low-pass cut-off frequency at 20 Hz and a high-pass cut off at 0.001Hz to remove the DC (0 Hz) noise. The R-waves in the ECG waveform were detected, and the corresponding time mapped SBP time series was generated. All data were re-sampled at 10 Hz using interpolation before further analyses were performed. Data pre-processing and analysis were performed using MATLAB 2009b (Mathworks Inc, MA, USA). Statistics were presented as means ± SD.

### Experimental protocol

The experiment was conducted in a sensory input reduced environment within an enclosed space of black drapes to remove all random visual stimuli. Measurements for height, weight and orthostatic correction for blood pressure were conducted prior to setup. Participants were set up for data acquisition prior to experimentation. After all the electrodes were placed, the participant was asked to sit quietly with a straight back and arms relaxed by the sides to verify the signal authenticity. The participants were required to be seated for 5 minutes, after which they were asked to stand (assistance was provided during the transfer from sit to stand) for 5 minutes with eyes open. They were instructed to make a passive transition from the seated to upright stance phase without altering their foot position. During the entire test duration, they were required to maintain eye-level gaze. The same 10-minute procedure was repeated with eyes closed with an imaginary eye-level gaze in the same position as with eyes open. The data with eyes closed were selected in the analysis, as postural sway increase and elevated levels of muscle activation were observed with the removal of visual input. The last 4-minute data during standing were used in the analysis.

Aggregate EMG was obtained by addition of rectified, zero–mean, EMG recordings from all individual leg muscles. The aggregate EMG measure has been used in exercise related studies [31] and was adopted similarly as a measure of total muscle activity. Data from five young (19–27 years), healthy, male participants were analyzed to check the applicability of the method.

## Results

### Simulations

**Figure 1:**
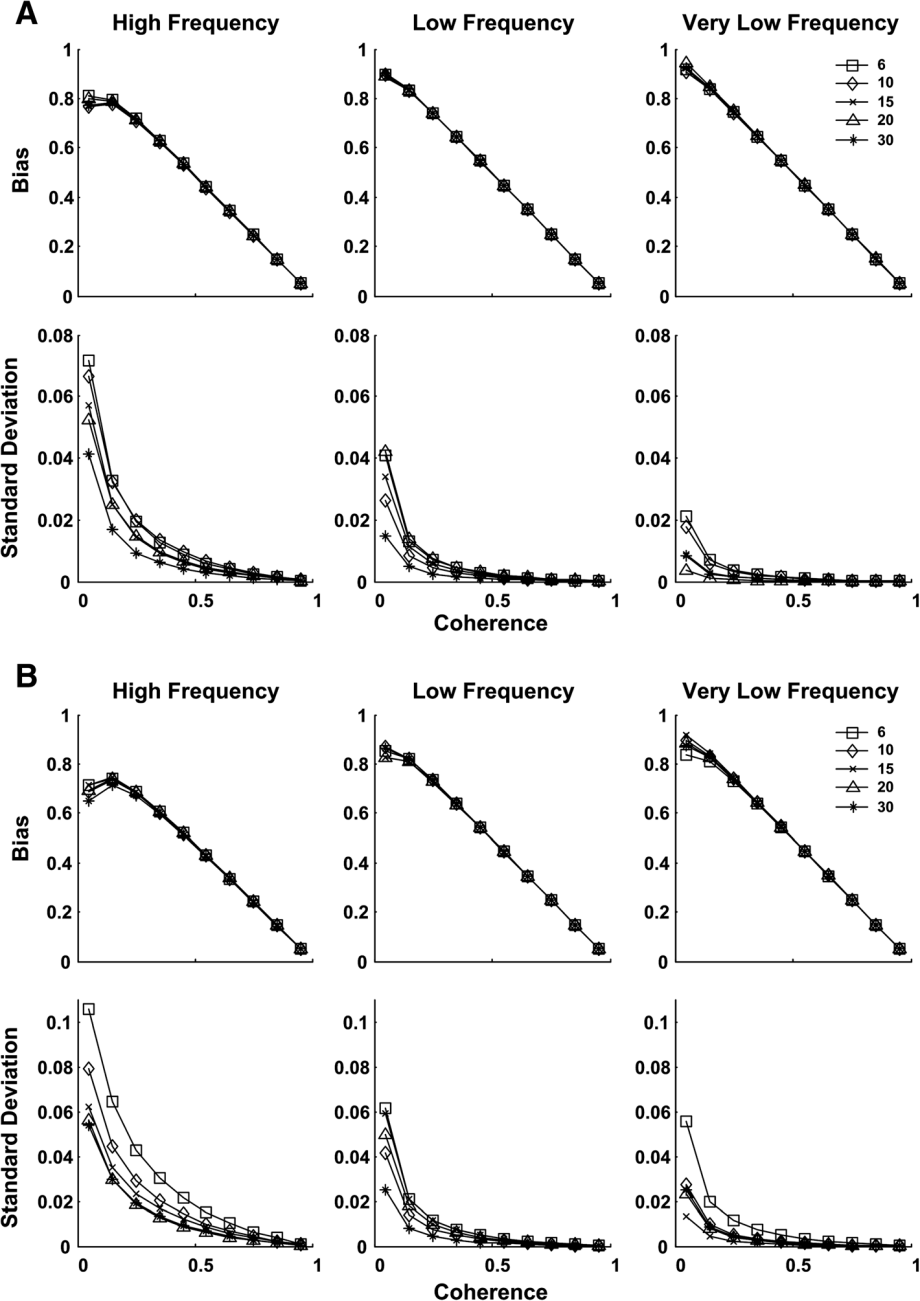
**Bias and standard deviation vs. coherence plots of the simulated electromyogram (EMG) signals based on (A): Laplacian and (B): Gaussian noises (*ω***_**0**_**=6 square; *ω***_**0**_**=10 diamond; *ω***_**0**_**=15 cross; *ω***_**0**_**= 20 triangle; *ω***_**0**_**=30 star).**

**Figure 2:**
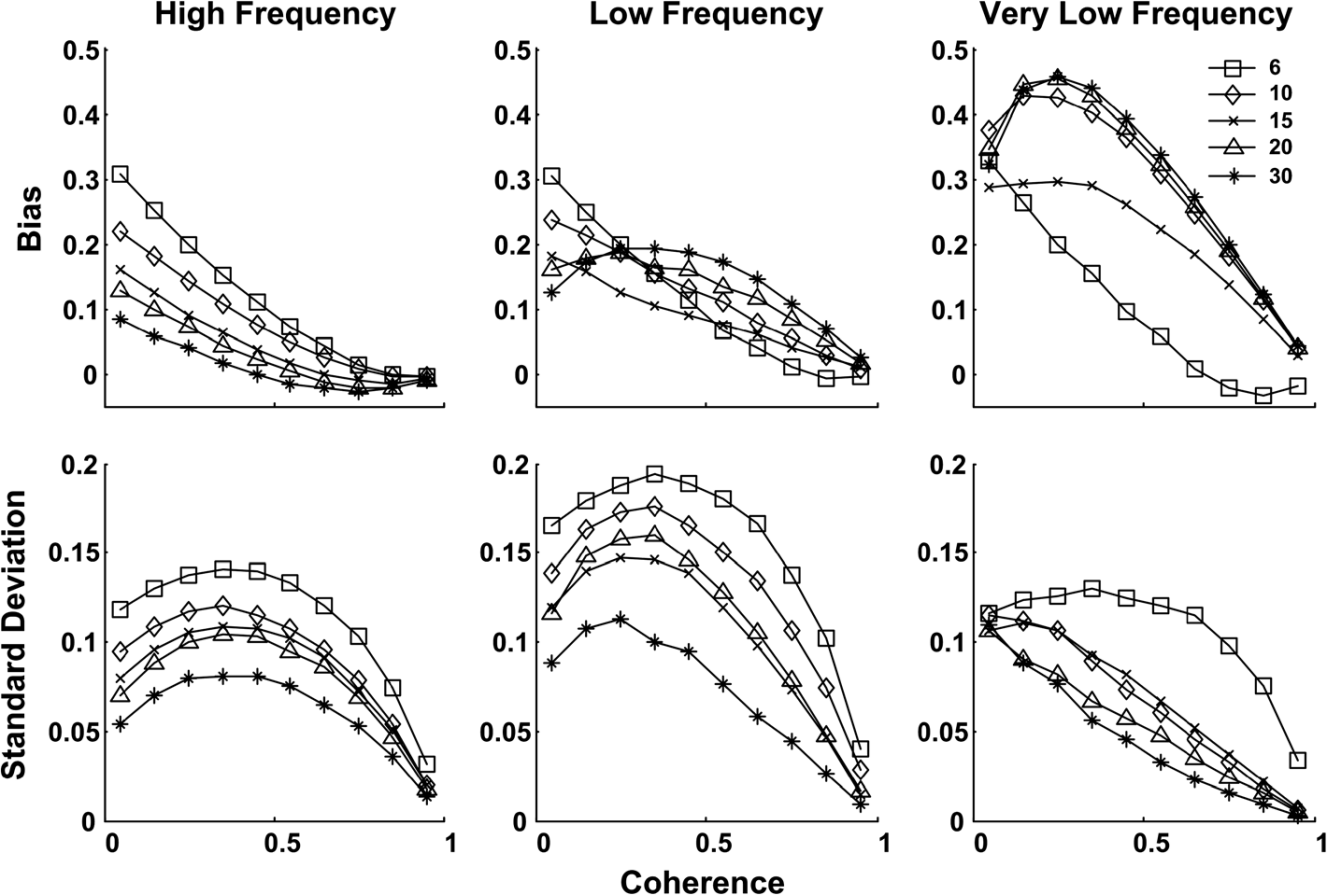
**Bias and standard deviation vs. coherence plots of the simulated systolic blood pressure (SBP) signals (*ω***_**0**_**=6 square; *ω***_**0**_**=10 diamond; *ω***_**0**_**=15 cross; *ω***_**0**_**=20 triangle; *ω***_**0**_**=30 star).**

For simulated EMG (Figure 1), bias and SD reduced with increasing levels of coherence. Different *ω*_0_ values yielded similar bias for EMG data. There was a steep fall in the value of SD for low coherence values which changed to a steady reduction for higher coherence. The Laplacian based EMG simulation yielded similar bias and slightly lower SD than the Gaussian simulation. With SBP (Figure 2) there was a trend towards a reduction in bias with increasing coherence levels. Within the range of interest for coherence in the current study (i.e., > ~0.3 (threshold)), *ω*_0_ = 6 showed overall small bias and consistent behavior across different frequency bands. Values of SD increased for lower coherence levels, and reduced for higher coherence levels after reaching a peak in between.

Based on the overall bias results, in conjunction with the limited number of data samples in the current study, we picked a small wavelet (i.e., *ω*_0_ =6) in the WTC analysis of real data. The threshold values of coherence between EMG and SBP were found to be at 0.3248 (HF), 0.3249 (LF), and 0.33 (VLF) for Gaussian EMG simulation and 0.3332 (HF), 0.3317 (LF), and 0.3318 (VLF) with Laplacian based simulation.

### Stand tests

**Figure 3:**
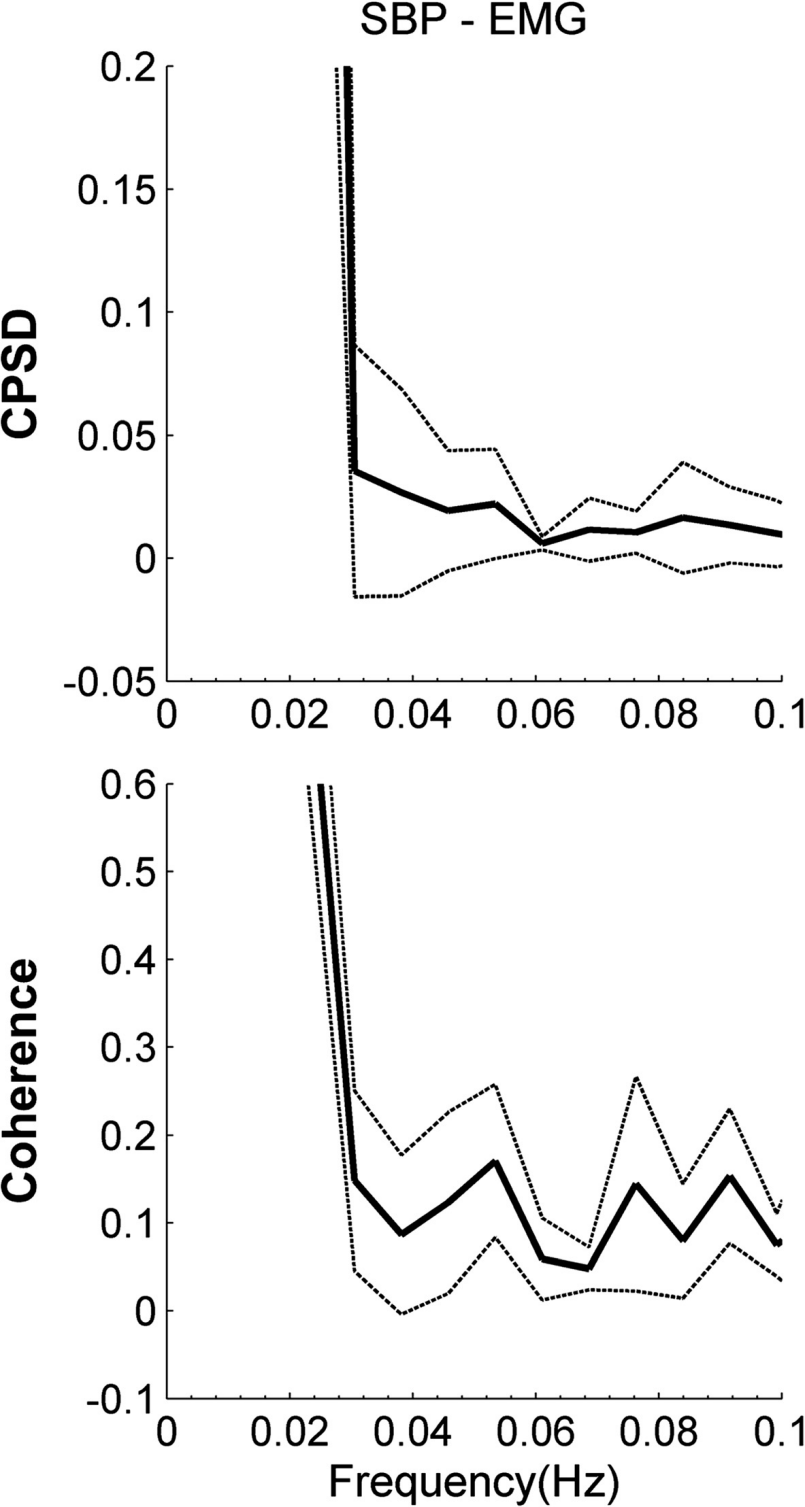
Average (bold lines) plus or minus one standard deviation (dash lines) cross-spectral power density (top) and coherence (bottom) estimates for young, healthy male participants (n = 5) between EMG and SBP signals.

The cross power spectral density (CPSD) analysis of the stand test data in the frequency range (0 – 0.1 Hz) showed peaks in the entire range (Figure 3) (coherence: 0.16 ± 0.05; power: 0.1 ± 0.06 Power/Hz). Both the spectral power and coherence between the signals showed peaks around 0.05 and 0.08 Hz. The results in Figure 3 are displayed in the frequency range (0.03 – 0.1 Hz) as the output had very less number of data points to provide interpretable results in the range < 0.03 Hz. The individual results for all participants’ data analysis are shown in the Table 1.

**Table 1:** Results from the analysis of real data of 5 male participants during quiet stance with eyes closed

	**CPSD**		**WTC**					
**Participant no.**	**Average value of coherence**	**Average power (power/Hz)**	**Average value of coherence**			**Percentage of time with significant interaction (%)**		
			HF	LF	VLF	HF	LF	VLF
1	0.15	0.04	0.31	0.39	0.52	37.9	67.2	100.0
2	0.14	0.04	0.34	0.32	0.42	53.8	36.5	86.4
3	0.13	0.19	0.30	0.25	0.39	35.5	31.1	75.2
4	0.16	0.12	0.30	0.40	0.40	38.0	84.0	100.0
5	0.25	0.11	0.39	0.27	0.50	67.1	23.7	100.0

The wavelet transform coherence (WTC) output is presented independently in the three frequency bands. The output for cross power spectral density (CPSD) analysis is averaged in 0.03 - 0.1 Hz frequency band.

**Figure 4:**
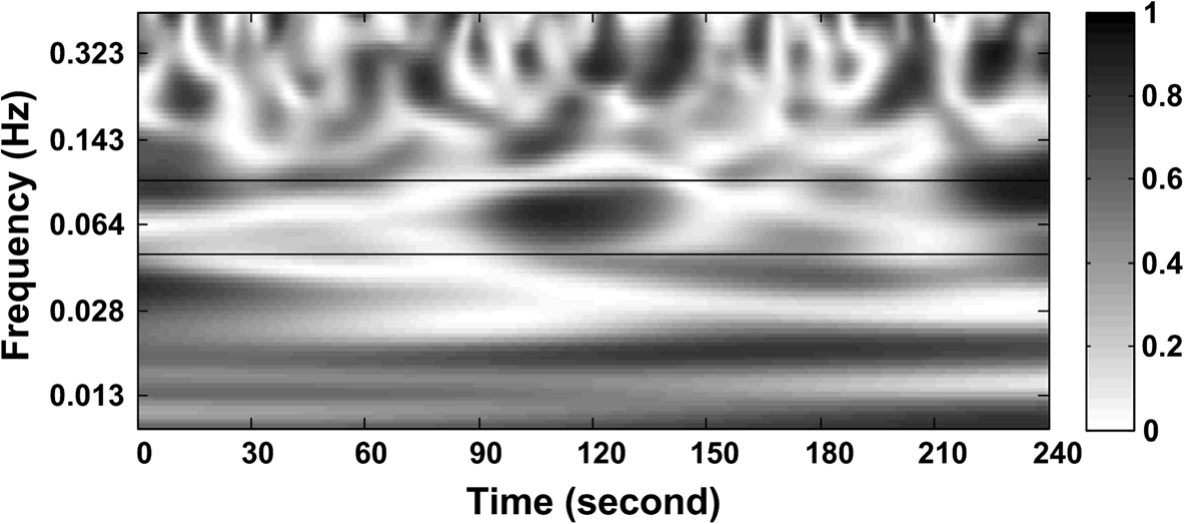
Time-frequency map of coherence between EMG and SBP obtained from one participant (number 2) using the wavelet transform coherence (WTC) analysis method.

**Figure 5:**
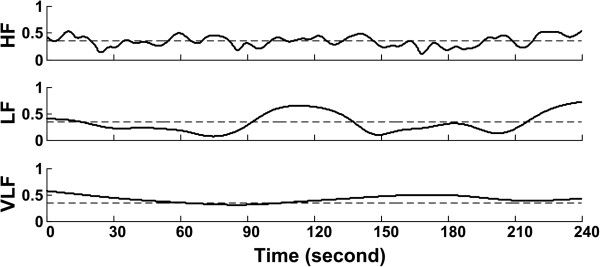
Three plots of band coherence obtained from averaging over corresponding frequency bands with the solid line representing the coherence between EMG and SBP and dashed straight line the significance level for the corresponding frequency band.

In the WTC analysis of the stand test data, the WTC threshold obtained from Laplacian EMG simulation were applied because of the low muscle contraction level during quiet standing [25]. The WTC estimator showed significantly high coherence in all three frequency bands. For the representative participant (number 2), the coherence time series was above significance levels for at least one of the three frequency bands at almost any time point in the whole duration under analysis (Figures 4 and 5). The coherence estimator (HF: 0.34; LF: 0.32; VLF: 0.42, averaged over 4 minutes) showed significant values for 53.8% (HF), 36.5% (LF), and 86.4% (VLF) of the whole time duration under consideration. The results from the analysis of all five participants’ data are included in Table 1. Averaging over all participants, the WTC estimator (HF: 0.33 ± 0.04; LF: 0.33 ± 0.07; VLF: 0.45 ± 0.06) detected significant coherence for 46.5 ± 13.6% (HF), 48.5 ± 25.8% (LF), 92.3 ± 11.2% (VLF) of the whole time duration under consideration.

## Discussion

The present study investigated the feasibility of the wavelet transform coherence method to detect the existence of a time varying relationship between fluctuations in blood pressure and muscle activation in the lower legs using simulated data and real physiological data from healthy young male individuals during quiet stance. The present study is one of the very few which have applied the WTC method for the analysis of relationships between non-stationary physiological signals, and the first to investigate the applicability in identifying the relationship between the BP and EMG signal pair. Classical coherence methods have often been used to find such relationships, but these methods assume a stationary nature of the signals. As BP and EMG signals have been identified to be non-stationary, these classical methods do not provide complete analyses. The WTC method provided a coherence output in the form of a time frequency map depicting corresponding coherence values for each time point in the frequency range of interest (Figures 4 and 5).

Classical coherence analysis similar to the previous research in our lab [5] was applied to the data to obtain the cross-spectral power and coherence variation with frequency (Figure 3). The plots show presence of peaks in the frequency range of 0.03 – 0.1 Hz. However, no acceptable information was received in the frequency range < 0.03 Hz, as the number of available data points was too low to obtain convincing results. The presence of peaks in the plots suggests that there exists a relationship between the signals which needs to be investigated further. These plots provided aggregate measure with frequency resolution, but no information was obtained regarding the change in the linear dependence with time. Hence this analysis only reiterated the previous results [5], but in this case showing the presence of coupling between the EMG and SBP signals during the normal condition. To further understand the time dependence of the signal interrelationship we therefore applied the WTC method to achieve both time and frequency resolution.

Time-frequency coherence maps were obtained for both simulated and human signals to quantify and validate the estimator in the HF (0.1 – 0.5 Hz), LF (0.05 – 0.1 Hz), and VLF (0.01 – 0.05 Hz) frequency bands. We chose to produce two representations of the coherence estimate (i) time-frequency maps of coherence value, (ii) coherence (average over a frequency range) with time. The simulated signal sets were derived from a band pass filtered noise signal as established in the literature [24,25,32]. The use of simulated data sets enables an objective evaluation of the analysis method. Statistical validation of the WTC estimator showed low values of bias and standard deviation within the range of interest for coherence for the simulated EMG (both Laplacian and Gaussian) and BP signal data sets. This signified an unbiased analysis of the inter signal coherence with minimal variance across the different analysis parameters. The threshold levels obtained from the simulated data sets are generic and independent of the source of data, and can be applied in further studies using such analysis. Note the WTC analysis was also validated on EMG data simulated from Gaussian noise in this study and the corresponding threshold of significant coherence can be utilized in future studies involving high muscle contraction levels (e.g., exercise).

Previous research with a similar application of WTC methods has shown comparable results with acceptable differences from the current study [19,20,23]. The differences in the values of the statistical measures and behavior with different values of the Morlet coefficient, *ω*_0_, were as expected based on the different types of signals under investigation. In particular, the current study investigated the EMG signal that has been shown to have a large random component [8]. The randomness in the signals, and the different frequency ranges of interest produced the observed differences and overall outcome of the analysis.

When applied to the real signal sets, the WTC method was successful in identifying zones of significant coherence between the two signals under investigation. Of particular interest was that the coherence time series was above significance (>T) for at least one of the three frequency bands at almost any time in the whole duration under analysis. This would indicate that there were changes in the spectral characteristics of the dependence between the two signals over time. The percentage time of significant coherence (Table 1) suggests that the signals were not interacting for the entire time duration, but only for a smaller section of time distributed over the entire duration under investigation.

The time-frequency representation of coherence also allowed for the assessment of the coupling between the two signals over different time scales (i.e., HF, LF, and VLF in the present study). The frequency ranges of interest were chosen in accordance to the results obtained in the Fourier cross-spectral analysis. While the origin of VLF interactions is not fully understood, significant coherence in HF (0.5 – 0.1 Hz) band may imply a respiration-driven coupling between postural control and BP regulation whereas a baroreflex-induced sympathetic outflow to the skeletal muscle may be characterized by the LF (0.1 – 0.05 Hz) coherence band. Further studies with more participants and experiment protocol including well controlled physiological perturbations are warranted to fully investigate the physiological relevance of wavelet coherence in these different frequency ranges.

The low values of coherence and spectral power obtained by classical analysis are due to the aggregation of the effect of changing dependence between the signals over time. On the other hand, the WTC analysis enabled us to find that there was a change in the dependence (coherence value) between the two signals which was not consistent throughout the time duration. Additionally, the WTC method found that the coupling between the signals increases and decreases in a non rhythmic pattern, suggesting that there is not likely a fixed pattern of coupling. On average over time the WTC estimator provided higher values of coherence than the classical coherence method, attributed to the characteristics of Morlet wavelet function applied to the signals under investigation. This implies that the results from the two methods indicate the same outcome, with the additional advantage of the time-frequency resolved output obtained from the WTC method.

As a validation study, the focus was to identify the overall coupling between lower limb muscle activity and the blood pressure on a system level. Therefore, an aggregate EMG was the reasonable choice in this context. However, summed EMG could potentially overlook the detailed roles of individual muscles in the cardio-postural interactions. Assessment on the relationships between individual lower limb muscle activation and BP regulation will be performed in subsequent studies. Moreover, investigations with more participants, hence greater statistical power, are warranted in the future to further compare the cardio-postural interactions under different conditions (e.g., eyes open vs. eyes closed) and/or between different groups (e.g., young vs. elderly).

## Conclusions

In the present study we established wavelet transform coherence analysis as a viable method for the analysis of the relationship between non-stationary blood pressure and calf muscle electromyography signals. The physiological relevance of the individual frequency bands was not evaluated, however; the time shifts in the individual signal characteristics may be related to the activity of other systems. Wavelet analysis provides a better and more powerful tool to investigate the hypothesized [5] bidirectional interaction between these two systems. Further in depth analysis to characterize the physiological dependence between cardiovascular regulation and lower leg muscle electromyographic activity is now required.

## Competing interests

The authors declare no competing interest (financial and non-financial) with any individual or organization.

## Authors’ contributions

AG: Created the theoretical framework for the validation of wavelet transform coherence method for use with EMG and BP signals. He also conducted the data acquisition and participant recruitment. DX: Assisted in the statistical analysis and creation of the manuscript and figures. APB: Assisted in the creation of the manuscript and the project idea along with the creation of the lab and its facilities enabling this study to be conducted at that time. All authors read and approved the final manuscript.
